# Vision in Futsal Players: Coordination and Reaction Time

**DOI:** 10.3390/ijerph18179069

**Published:** 2021-08-27

**Authors:** Henrique Nascimento, Cristina Alvarez-Peregrina, Clara Martinez-Perez, Miguel Ángel Sánchez-Tena

**Affiliations:** 1 ISEC Lisboa, Instituto de Educação e Ciência de Lisboa, 1750-179 Lisboa, Portugal; henrique.nascimento@iseclisboa.pt (H.N.); masancheztena@ucm.es (M.Á.S.-T.); 2 Department of Pharmacy, Biotechnology, Nutrition, Optics and Optometry, Faculty of Biomedical and Health Science, Universidad Europea de Madrid, 28670 Madrid, Spain; cristina.alvarez@universidadeuropea.es; 3 Department of Optometry and Vision, Faculty of Optics and Optometry, Universidad Complutense de Madrid, 28037 Madrid, Spain

**Keywords:** reaction time, visual coordination, visual reaction

## Abstract

Background: Coordination and reaction time are relevant aspects of a sport’s competitive performance within teams. The aim of this study was to explore if a group of futsal players, in a laboratory context, would present better results from actions where vision is prevalent compared to a control group without contact with futsal or any other sport. Methods: The digital system of the COI- SV software was used; six tests were selected, related to coordination (“Eye/hand coordination”; “Coordination and identification”) and reaction time (“Anticipation Time”; “Peripheral response”; “reaction time”; “Visual memory”). Results: Of all the tests performed, only in the anticipation time test did the futsal players obtain better results than the control group. The average time of the failures was lower in relation to the control group. In the others, no differences were found between the two groups. Conclusions: The futsal players did not perform better than the control group in most of the tests carried out, except in the “anticipation time”. Therefore, visual training maybe necessary to improve visual skills and sports performance.

## 1. Introduction

The field of sports optometry is relatively new; therefore, it requires that more scientific studies be developed to prove the effectiveness of visual training in sports [1].

Futsal is a sport that was born in the 1930s (s. XX), due to the lack of spaces for the practice of soccer in Uruguay, eventually being adapted to basketball courts and other halls [2]. Futsal, in countries that have football as their national sport, is usually the second most important sport and has the largest number of practitioners. The principles of futsal are like other sports that practice in the same kind of courts like basketball, handball, and roller hockey. Therefore, when analyzing issues related to futsal, not related to vision, they may be applied to the abovementioned sports [3].

It is quite common to say that football/futsal is not taught. This is because the practice of futsal depends on a natural tendency, vocation, and skill of the practitioner. However, the development of motor abilities is essential, and learning and training techniques aim to develop the skills of the player improving its natural talents [4].

Regarding vision, there is no standard for football/futsal and the nature of the relationship between performance and vision development is unknown [2,5]. However, there are studies that conclude that visual training improves visual skills, which in turn are transmitted to sports performance [6]. As early as 1988, the visual skills associated with sports performance were analyzed. Thus, a battery of tests that approximate the visual abilities observed in sport, titled Pacific Sports Visual Performance Profile (PSVPP), was used in order to generate normative data. This study offered normative data for non-athletes on standardized optometric tests and provided a basis for future work in vision and athletics [7].

In the study by Formenti et al. [8], they analyzed the effect of sports vision training programs for six weeks. The results showed that vision training improves cognitive skills in a non-sport-specific context (both generic and with specific motor actions), but appears to be less effective in improving sport-specific skills. This suggests that the environment where the exercises are performed plays a key role in improving perception and action on sport-specific skills, supporting the ecological approach to sports learning.

According to Welford [9], three main mechanisms govern the human sensorimotor system: perception, decision, and execution/control. The process is the perception of outside information; a decision based on what is perceived; and execution of the action according to what was decided—analysis of the result involves the sensory perception mechanism, decision-making mechanism, and execution mechanism. Later, in the study carried out by Land, it was corroborated that the visually mediated actions are based on three systems: the gaze system responsible for locating and fixing the relevant objects for the task, the motor system of the extremities to carry out the task, and the visual system to supply information to the other two. These three systems are under the supervision of a fourth system, which is the outline system, which specifies the current task and plans the general sequence of actions. These four systems have separate but interconnected cortical representations [10].

Athletic performance is highly dependent on vision [11]. There are different views on the most critical factors in athletic performance, but few disagree that vision is one of them.

The late Blanton Long Collier, a football coach who trained at the University of Kentucky between 1954 and 1961 and at the Cleveland Browns of the National Football League between 1963 and 1970, said: “eyes lead the body”. He was one of the first coaches to not only recognize that vision is critical in performance, but also that the quality of vision differs from player to player [12]. Recognizing this importance, an interdisciplinary analysis of the structure and the participants in its competitive component is necessary, so that we can do a job that improves the player’s natural abilities [13].

The objective of this study is to analyze whether futsal players have more developed visual coordination and reaction time skills compared to a control group, without contact with competitive sports.

## 2. Materials and Methods

A cross-sectional study was conducted to compare the visual skills of players from a futsal team with a control group. Inclusion criteria for both groups were aged between 18 and 30, visual acuity between 0.0 and 0.1 (LogMAR), binocular vision with vergences within normal limits and stereopsis among 40 and the 20’ of arc. All participants were male, and the study took place between the months of September and November 2019 at the High-Performance Center in Sports Vision at ISEC Lisbon. Ten futsal athletes from the Portuguese senior championship the 2° division were randomly chosen. As a control group, thirty-five individuals in the same age group without contact with the sport were also chosen. Visual efficacy of all participants was evaluated with the digital system COI-Sport Vision (Centro de Optometría Internacional, Madrid, Spain), choosing the tests that measure the most relevant visual abilities for futsal [12,14]. The reliability of this software is (α = 0.93) [15]. All athletes were previously introduced to each of the tests and had a learning time, in order to ensure good repeatability of the tests and that there was no bias in the results. At the same time, during the tests all the athletes could take the tests in the time they needed, until the test ended once the tests established by the software in each test had been carried out. Peripheral vision is truly relevant in futsal practice [16]; one of the ways to train and improve it is by using eye–hand coordination exercises, which is why this system was chosen. 

The order of conducting the tests was as follows:

1. Anticipation time: (reaction time): A moving object (a red ball) moves across the screen. The subject must touch the screen just as the ball passes through a rectangular area that can be in any position on the screen. The aim is that the subject could predict when the ball is going to pass through the rectangle, pressing with his/her hands or feet on the sensors to stop it. The test was performed with the dominant hand of all subjects.
2. Eye–hand coordination, coordinative reaction speed: The main purpose of this test is to train eye–hand and eye–foot coordination. The subject should press the end of the arrow with their dominant hand, which varies in orientation. The software gives information about the percentage of correct answers and how long does it take to the subject in seconds.
3. Coordination and identification: The subject should touch the red ball that moves across the screen. Along with the red ball, multiple balls of different colors appear and move on the screen as a distraction.
4. Attention and peripheral response PAT: In this test, a central circle shows a flashing light. At the ends of eight arms, other lights appear randomly. As soon as the center light and one of the peripheral lights are the same color, the subject must touch the arm light with the same color before it changes again.
5. Reaction capacity (penalty): In this test, the software measures the reaction time to the appearance of a stimulus with a certain directionality. The subject has to anticipate the movement of the ball moving across the screen and hit it through the clicker with their feet.
6. Visual memory (Tic Tac Toe): The subject has to remember the positions of various symbols that are changing position in a 3 × 3 grid.

### 2.1. Statistical Analysis

Statistical analysis was conducted using the SPSS 25.0 software by (IBM Corp.: Armonk, NY, USA). Concerning the parametric distribution of the variables, the Shapiro-Wilk test (n < 50) was used. To determine the effect size, Cohen’s *d* measure has been used (0.2 indicates a small effect size, 0.5 medium magnitude and 0.8 indicates a high magnitude effect). To check if there was a significant association between the continuous quantitative variables, the Pearson test was used if they had a normal distribution or the Spearman test if the distribution was not normal. The Mann–Whitney U test was used to compare the two groups. At the same time, to assess statistical significance, a cut-off point of *p* ≤ 0.05 was considered. 

### 2.2. Ethical Approval

This study was approved by the ethics commission of the General Directorate for Research and Development (DGID) of Instituto Superior de Educação e Ciências (ISEC) Lisbon, Portugal. The ethical approval number is 01/27052020.

## 3. Results

The total number of participants was 45 (10 futsal players and 35 control group; *d* = 0.42), hence representing small effect size, according to Cohen’s *d* measure. The ages were between 18 and 30 years old, the average age and standard deviation of futsal players was 22.8 ± 3.6 years, and control group 24.5 ± 2.7 years.

### 3.1. The Anticipation Time

**Figure 1:**
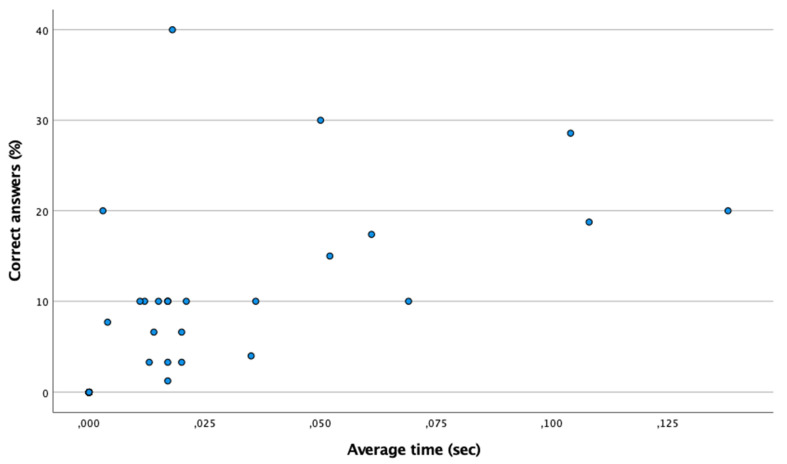
Positive linear relationship between the percentage of correct answers and the mean time in the anticipation time test. The point (0,0) means that there is no correct answer.

**Figure 2:**
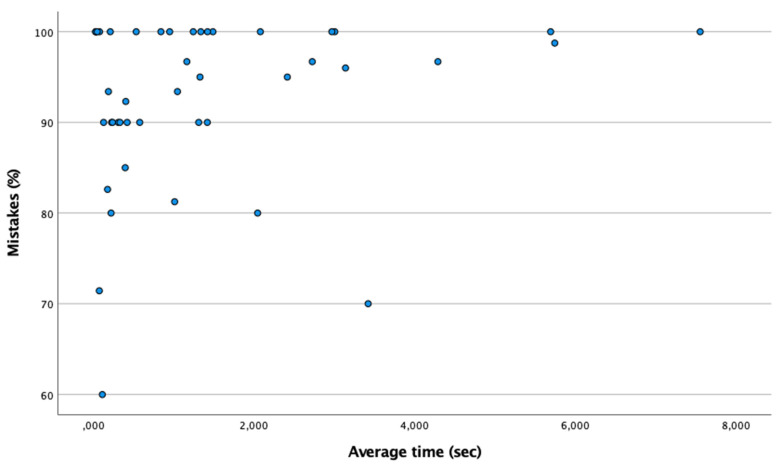
Absence of relationship between the percentage of failures and the mean time in the anticipation time test.

Table 1 shows the percentage and mean time of hits and misses the anticipation time. According to the Shapiro–Wilk test (n < 50), a non-normal distribution was found (*p* < 0.05). Therefore, using the Spearman test, Figure 1 shows the existence of a moderate and positive correlation between the time in reaching correct answers in the percentage of correct answers (Spearman: ρ = 0.877; *p* ≤ 0.001). However, as shown in Figure 2, there is no association between mean time and percentage of mistakes (Spearman: ρ = 0.170; *p* > 0.05). Therefore, there are no differences in the mean hit time between the controls and the futsal players (*p* > 0.05). However, the mean time to failures of the group of futsal players is lower than that of the control group (*p* = 0.015). 

### 3.2. Eye–Hand Coordination Test

**Figure 3:**
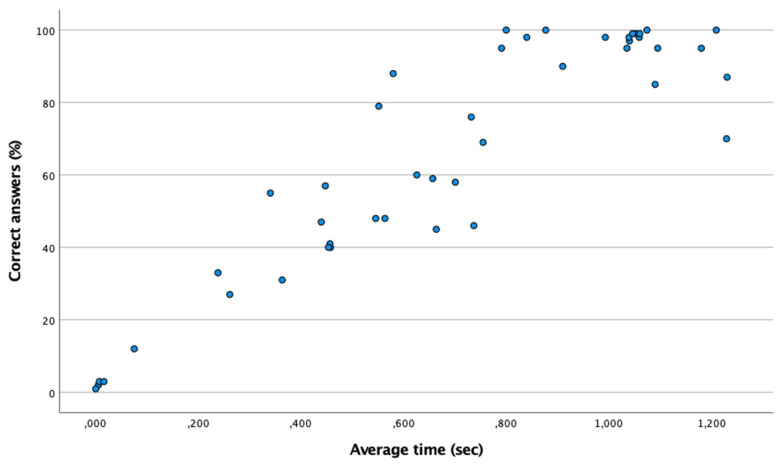
Positive linear relationship between the percentage of correct answers and the average time in the eye–hand coordination test. The point (0,0) means that there is no correct answer.

Table 2 shows the percentage of correct answers and the average time of the eye–hand coordination test. According to the Shapiro–Wilk test (n < 50), it is a non-normal distribution (*p* < 0.05), so the Spearman test has been used. Figure 3 shows the existence of a moderate and positive correlation between the time and the percentage of correct answers (Spearman: ρ = 0.838; *p* ≤ 0.001). Nonetheless, the time of the futsal group is similar to that of the control group (*p* > 0.05).

### 3.3. Coordination and Identification Test

**Figure 4:**
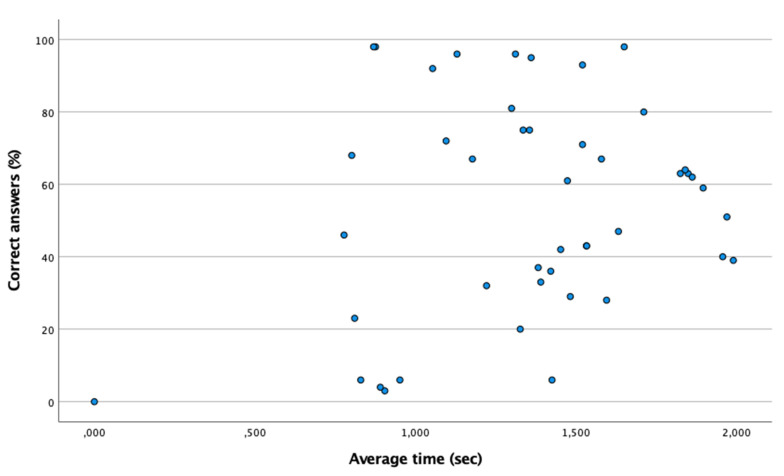
Absence of relationship between the percentage of correct answers and the mean time in the coordination and identification test. The point (0,0) means that there is no correct answer.

Table 3 shows the percentage of correct answers and the average time of the coordination and identification test. According to the Shapiro–Wilk test (n < 50), it is a non-normal distribution (*p* < 0.05). Using the Spearman test, in Figure 4 no association was found between the time and the percentage of correct answers (Spearman: ρ = 0.086; *p* > 0.05). In turn, the mean time of the group of futsal players is similar to that of the control group (*p* > 0.05).

### 3.4. Attention and Peripheral Response (PAT Test)

**Figure 5:**
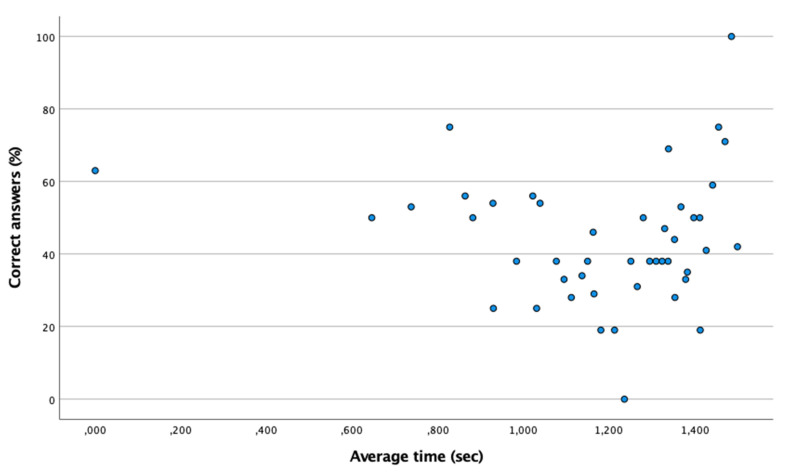
Absence of relationship between the percentage of correct answers and the mean time in the attention and peripheral response.

Table 4 shows the percentage of correct answers, the average time and the lateral preference of the attention and Peripheral Response PAT test. According to the Shapiro–Wilk test (n < 50), time is a variable with a non-normal distribution (*p* < 0.05). The rest of the variables have normal distributions (*p* > 0.05). As shown in Figure 5, there is no significant association between time of answer and the percentage of correct answers (Pearson: r= −0.069; *p* > 0.05/ Spearman ρ = 0.036; *p* > 0.05). Therefore, the mean time of the futsal players is similar to that of the control group (*p* > 0.05).

When analyzing lateral preference of the visual field in the subjects, a significant association was found between the time, the percentage of correct answers, and the lateral preference left (Pearson: r = 0.457; *p* < 0.05/ Spearman: ρ = 0.364; *p* < 0.05). However, the lateral preference of the futsal players is similar to that of the control group (*p* > 0.05). 

### 3.5. Reaction Capacity (Penalty)

**Figure 6:**
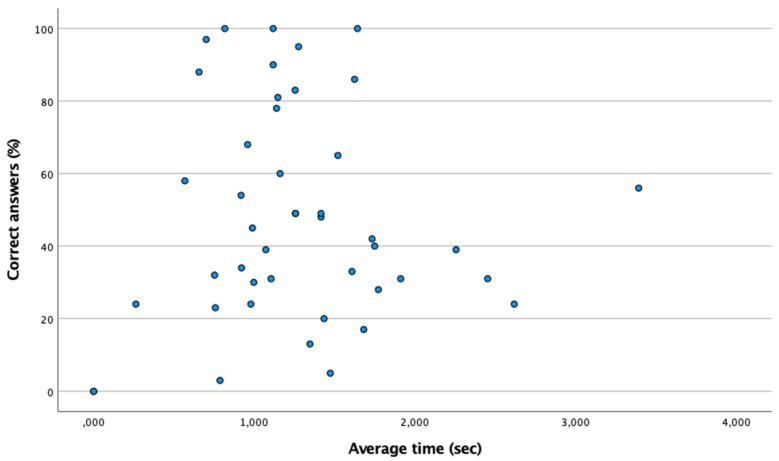
Absence of relationship between the percentage of correct answers and the mean time in the reaction capacity. The point (0,0) means that there is no correct answer.

Table 5 shows the percentage of correct answers and the average time of the reaction capacity (penalty). The Shapiro–Wilk test (n < 50) indicates a non-normal distribution (*p* < 0.05). Therefore, using the Spearman test, Figure 6 shows that there is no association between the time and the percentage of correct answers (Spearman: ρ = 0.002; *p* > 0.05). In turn, the mean time of the futsal players is similar to that of the control group (*p* > 0.05). 

### 3.6. Visual Memory Test

**Figure 7:**
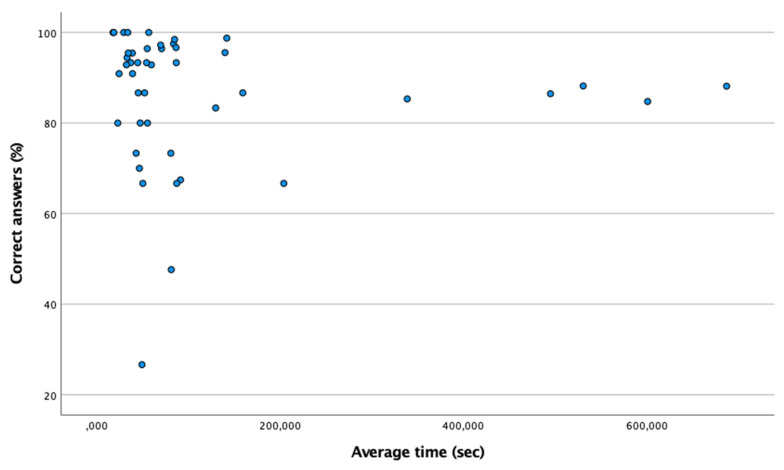
Absence of relationship between the percentage of correct answers and the mean time in the visual memory test.

Table 6 shows the percentage of correct answers and the average time of the visual memory test. According to the Shapiro–Wilk test (n < 50), it is a non-normal distribution (*p* < 0.05). Therefore, using the Spearman test, Figure 7 shows that there is no association between the total time and the percentage of correct answers (Spearman: ρ = −0.262; *p* > 0.05). In turn, the mean time of the futsal players is similar to that of the control group (*p* > 0.05). 

## 4. Discussion

It was not demonstrated in this study that by having a competitive sport activity, it is possible to develop and improve visual skills, which in turn can contribute to a better sport performance. The most important visual skills in sports are visual acuity (static and dynamic, and central and peripheral), refractive error, accommodation (amplitude; far near; relationship with convergence), eye movements, binocularity, visual perception, and visuomotor abilities [17]. 

In the article by Jorge et al. [14], they used the same software as in our study, COI-SV, for the analysis of dynamic visual acuity where 147 high-level futsal players were analyzed. In this article, they concluded that dynamic visual acuity varies by playing position. On the contrary, to complete what these researchers concluded, in this article it was intended to demonstrate if there was a difference between other skills such as anticipation time, eye–hand coordination, coordinative reaction speed, coordination and identification, attention and peripheral response, reaction capacity and visual memory (Tic Tac Toe). Our results agree with theirs, demonstrating the importance of vision on the field of play. In turn, both results demonstrate the validity and usefulness of sports vision software to improve performance on the playing field.

In general, eye movements, for visual information sources, precede motor actions that are supervised by the eyes. In fact, a task that requires sequential fixation requires a selection of the important in relation to the accessory [10].

However, a recent study showed that visual–motor skills play a fundamental role in sports performance suggesting that sensorimotor scans can be a useful tool in the observation of players [8]. Proprioception is a relevant aspect in this entire process, since it plays an important role in motor planning, as well as in the quick connection with the adaptation mechanisms for effecting performance changes during an execution [18]. Despite the fact that in our results, we have not found differences in the coordination between the futsal players and the control group, if we base on the studies mentioned above, we can come to think that a training with a longer duration can improve the coordination of footballers.

There is little information about which visual skills are important for an athlete in a specific sport and about what capacity is needed to ensure consistency. As interest in sports vision is increasing, practitioners are adopting a wide diversity of methods to assess the athlete’s visual skills. To improve communication between sports vision practitioners and researchers, standardized test batteries have been developed, designed to assess the different visual skills to maximized sports performance. Referred to as PSVPP (Pacific Sports Visual Performance Profile), a battery of 23 tests were designed to evaluate visual performance related to athletic competition. In addition to the classic tests of visual acuity, refractive condition, and eye health, this battery includes tests of visual performance related to the needs of tasks in different sports [19]. Thus, when comparing it with our study, we can see that since the 20th century a battery of tests was carried out to improve the vision of athletes, which has served as a guide for the development of the software that exists today.

The PSVPP includes several tests, with that on reaction time being one of the most important. Reaction time measures how fast an athlete reacts to a visual stimulus that reaches the sensory system before the motor response begins [20]. It can also be defined as the rate of preparation necessary to move, that is, the time before the action. It has an amplitude in milliseconds (ms), manifesting with different values according to the sensory system [21]. The tactile reaction time is approximately 110 (ms), the auditory is about 150 (ms) and the visual is just about 200 (ms). In the reaction time process, the stimulus via afferent reaches the primary somatosensory cortex and the posterior parietal cortex, this sensorimotor integration sends the information to area 6 of the motor cortex, where movement planning takes place. This moment of the reaction time is known as pre-motor. In the motor period, the information from area 6 is sent to area 4 of the motor cortex to generate the intention to start the movement, and the cerebellum has an important role in guiding this future action [22]. Depending on the quality of cortical excitability, the reaction time can be performed at a faster or slower speed.

Reaction time is a difficult motor skill to be trained, as it depends on the athlete’s genetics. However, it can be improved by 10–20% with visual training [23]. Professional athletes have faster reaction times than beginners or untrained [23]. When the athlete is very focused on performing an activity, his reaction time is faster [24]. Other factors influence the speed of reaction time and, consequently, the speed of motor response, such as age (20 to 30 years is the peak), the type of training practiced by the athlete, their physical conditioning, the individual’s level of fatigue and the cognitive level (the more intelligent, the faster reaction time) [25,26]. The reaction time is important for the practice of several sports and particularly important for futsal [27]. This agrees with the results of our study, where it has been obtained that after vision training, reaction time is better in futsal players than in the control group. Therefore, it improves the results on the playing field.

For all this, the vision must accompany the evolution of the athlete so that he can be increasingly competent in his role integrated into a team. As has been said, in practically all athletes, there is an innate part and a trainable part, which is the same as saying that it can be improved. When the athlete reaches a stage of high competition, and in exercises in which his inaptitude or his refined technique is not at stake, he is not able to rise above non-athletes, so something is not being done well in relation to his training. We verified in the results of this study, that in relation to the reaction time to different stimuli and situations outside the game environment, athletes did not clearly outperform non-athletes. On the other hand, since futsal is an activity that depends a lot on the reaction/action, it would be normal for the athletes to perform better than that seen in these results [28]. As visual training increases and develops all patterns of visual behavior and as psychologists estimate that 80% of the information we get from our external environment is through our visual pattern, we can consider this to be the most important part of the behavior pattern of the athlete [29]. Therefore, it is foreseeable that when they are part of visual training programs that improve their abilities, they can thus improve their individual and collective sports performance. Furthermore, the development of visual skills may also benefit athletes in preventing injuries [30]. There is ample evidence that training of visual skills administered in a definitive approach and on an individual basis following particular guidelines can lead to an improved performance in various aspects of sports eventually leading to a top-level performance desired by most athletes [31].

Regarding the limitations of our study, the software used in our study has been used in another published research [14]. The decision to use this software is that COI-SV can perform both diagnostic tests and optometric treatments with a specific focus on sports vision. Without limitation of distance, it allows to work from next to the touch screen up to 15 m. In addition, all the programs have different levels of difficulty and allow us to work by quadrants of the visual field. At the same time, it is one of the few existing software that allows us to analyze various visual abilities as well as perform both visual examination and training.

## 5. Conclusions

In this study, we concluded that the futsal players did not present better visual skills than the control group, except in “eye/hand coordination”. This makes sense if we accept better coordination in an athlete as normal, in which the results were superior.

It has been proven that being a competitive player with daily sports training and with innate conditions for practicing this sport does not imply that, in terms of vision, performance is superior to a control group without contact with competitive sports.

Knowing that vision has a lot of relevance in futsal game actions and that visual skills can be developed through sports visual training programs, it is important to continue making studies that prove that improving these skills can lead to better sports performance, thus being able to motivate athletes, coaches, and managers to adopt this type of training.

## Figures and Tables

**Table 1:** Results of Anticipation time test.

	Futsal Players				Control				
	Mean	SD	Median	CI	Mean	SD	Median	CI	*p*-Value
Percentage of correct answers (%)	6.2	9.1	1.6	−0.3–12.7	7.7	9.7	4.0	4.2–11.1	0.620
Time to reach the correct answer (sec)	0.02	0.03	0.01	0.00–0.04	0.02	0.03	0.01	0.01–0.03	0.459
Percentage of mistakes (%)	93.8	9.1	98.3	87.3–100.3	92.5	9.4	92.5	88.9–95.7	0.600
Time to reach a mistake (sec)	0.71	1.15	0.15	−0.11–1.53	1.64	1.81	1.04	0.96–2.28	0.015

SD: standard deviation; CI: Confidence interval; sec: seconds.

**Table 2:** Results of eye–hand coordination test.

	Futsal Players				Control				
	Mean	SD	Median	CI	Mean	SD	Median	CI	*p*-Value
Percentage of correct answers (%)	67.0	35.2	77.0	41.8–92.2	65.6	31.8	70.0	54.7–76.5	1.000
Time to reach the correct answer (sec)	0.68	0.35	0.75	0.43–0.93	0.70	0.37	0.73	0.57–0.83	0.819

SD: standard deviation; CI: Confidence interval; sec: seconds.

**Table 3:** Results of coordination and identification test.

	Futsal Players				Control				
	Mean	SD	Median	CI	Mean	SD	Median	CI	*p*-Value
Percentage of correct answers (%)	64.6	32.1	71.5	41.6–87.6	50.3	28.6	47.0	40.5–60.1	0.162
Time to reach the correct answer (sec)	1.24	0.31	1.31	1.01–1.46	1.39	0.43	1.45	1.24–1.53	0.124

SD: standard deviation; CI: Confidence interval; sec: seconds.

**Table 4:** Results of Peripheral Response PAT test.

	Futsal Players				Control				
	Mean	SD	Median	CI	Mean	SD	Median	CI	*p*-Value
Percentage of correct answers (%)	39.7	11.9	36.5	31.1–48.2	44.9	19.2	44.0	38.3–51.5	0.311
Time to reach the correct answer (sec)	1.02	0.41	1.12	0.73–1.31	1.22	0.22	1.29	1.15–1.30	0.111
Percentage of correct answer in RIGHT side (%)	28.7	20.6	29.0	13.9–43.5	46.1	25.0	50.0	37.5–54.7	0.057
Percentage of correct answer in LEFT side (%)	41.5	17.7	42.5	28.85–54.14	43.4	24.00	42.0	35.19–51.67	0.989

SD: standard deviation; CI: Confidence interval; sec: seconds.

**Table 5:** Results of the reaction capacity test.

	Futsal Players				Control				
	Mean	SD	Median	CI	Mean	SD	Median	CI	*p*-Value
Percentage of correct answers (%)	51.4	30.2	44.5	29.8–73.0	47.1	29.6	42.0	36.9–57.2	0.697
Time to reach the correct answer (sec)	1.61	0.82	1.56	1.02–2.20	1.17	0.54	1.14	0.98–1.35	0.111

SD: standard deviation; CI: Confidence interval; sec: seconds.

**Table 6:** Results of the visual memory test.

	Futsal Players				Control				
	Mean	SD	Median	CI	Mean	SD	Median	CI	*p*-Value
Percentage of correct answers (%)	87.9	11.7	90.8	79.5–96.2	85.7	15.9	90.9	80.3–91.1	0.717
Time to reach the correct answer (sec)	127.79	150.35	75.78	20.23–234.34	114.09	161.65	54.34	58.56–169.62	0.366

SD: standard deviation; CI: Confidence interval; sec: seconds.

## Data Availability

Not applicable.
